# Supplementary material for: The Influence of Envelope C-Terminus Amino Acid Composition on the Ratio of Cell-Free to Cell-Cell Transmission for Bovine Foamy Virus
Source: Viruses. 2019 Jan 31;11(2):130. doi: 10.3390/v11020130 (PMC6410131; doi:10.3390/v11020130)
Supplement: Supplementary file 1 [file viruses-11-00130-s001.pdf]

**Table S1.** The mutation amino acid (aa) sites in coding region of Cell-free BFV.

| | Gag | | | Pol | | | Env | |
|---|---|---|---|---|---|---|---|---|
| **AA site** | BFV-B | BFV-Z1 | **AA site** | BFV-B | BFV-Z1 | **AA site** | BFV-B | BFV-Z1 |
| **78** | P | T | **16** | P | L | **42** | D | N |
| **176→** | PLPAP | ...... | **18** | S | G | **55** | V | I |
| **189** | PIGPP | ...... | **57** | Y | C | **134** | P | H |
| | PPAA | ..... | **111** | I | V | **816** | H | Y |
| **440** | S | F | **118** | S | P | **823** | P | S |
| | | | **174** | G | D | **898** | E | K |
| | **Tas** | | **315** | V | M | **976** | R | K |
| **AA site** | BFV-B | BFV-Z1 | **344** | K | R | **978** | L | S |
| **184** | C | R | **862** | T | N | | | |
| | | | **901** | I | V | | | |
| | **Bet** | | **974** | N | S | | | |
| **AA site** | BFV-B | BFV-Z1 | **1080** | P | L | | | |
| **313** | S | G | **1179** | H | Y | | | |
| **366** | E | K | | | | | | |
| **392** | R | H | | | | | | |

**Table S2.** The mutation sites in noncoding region of Cell-free BFV.

| | 5'LTR | | | 3'LTR | |
|---|---|---|---|---|---|
| **position** | BFV-B | BFV-Z1 | **position** | BFV-B | BFV-Z1 |
| **76** | A | G | **10803** | A | G |
| **340** | G | A | **10962** | G | A |
| **662** | A | G | **11041** | G | A |
| **695** | A | G | **11164** | G | A |
| **729** | G | A | **11308** | C | T |
| **820** | T | C | **11396** | A | G |
| **833** | G | A | **11430** | A | G |

| | | | | | |
|---|---|---|---|---|---|
| **908** | G | A | **11438** | C | A |
| **934** | G | A | **11534** | G | A |
| **971** | C | T | **11609** | G | A |
| **985** | A | C | **11635** | G | A |
| **993-994** | .. | CT | **11646** | T | C |
| **1021** | T | A | **11683** | A | G |
| **1107** | G | A | **11686** | A | G |
| **1118** | C | T | **11693-11694** | .. | CT |
| **1131** | C | T | **11722** | T | A |
| **1160** | T | C | **11808** | G | A |
| **1161** | C | T | **11819** | C | T |
| **1249** | G | A | **11861** | T | C |
| | | | **11862** | C | T |
| | **IP** | | **11927** | T | C |
| **position** | BFV-B | BFV-Z1 | | | |
| **9251** | A | G | | | |
| **9315** | G | A | | | |
